# Electrooptical Determination of Polarizability for On-Line Viability and Vitality Quantification of *Lactobacillus plantarum* Cultures

**DOI:** 10.3389/fbioe.2018.00188

**Published:** 2018-12-04

**Authors:** Klaus Pellicer-Alborch, Alexander Angersbach, Peter Neubauer, Stefan Junne

**Affiliations:** ^1^Chair of Bioprocess Engineering, Department of Biotechnology, Technische Universität Berlin, Berlin, Germany; ^2^EloSystems GbR, Berlin, Germany

**Keywords:** probiotics, polarizability, *Lactobacillus plantarum*, viability analysis, freeze–drying, cell length quantification, lactic acid bacteria, process analytical technology

## Abstract

The rapid assessment of cell viability is crucial for process optimization, e.g., during media selection, determination of optimal environmental growth conditions and for quality control. In the present study, the cells' electric anisotropy of polarizability (AP) as well as the mean cell length in *Lactobacillus plantarum* batch and fed-batch fermentations were monitored with electrooptical measurements coupled to fully automated sample preparation. It was examined, whether this measurement can be related to the cells' metabolic activity, and thus represents a suitable process analytical technology. It is demonstrated that the AP is an early indicator to distinguish between suitable and unsuitable growth conditions in case of a poor energy regeneration or cell membrane defects in *L. plantarum* batch and fed-batch cultivations. It was shown that the applied method allowed the monitoring of physiological and morphological changes of cells in various growth phases in response to a low pH-value, substrate concentration changes, temperature alterations, exposure to air and nutrient limitation. An optimal range for growth in batch mode was achieved, if the AP remained above 25·10^−28^ F·m^2^ and the mean cell length at ~2.5 μm. It was further investigated, in which way the AP develops after freeze-drying of samples, which were taken in different cultivation phases. It was found that the AP increased most rapidly in resuspended samples from the retardation and late stationary phases, while samples from the early stationary phase recovered slowly. Electrooptical measurements provide valuable information about the physiologic and morphologic state of *L. plantarum* cells, e.g., when applied as starter cultures or as probiotic compounds.

## Introduction

Lactic acid bacteria are applied for food preservation, but more importantly for yogurt and probiotics production. *Lactobacillus plantarum* plays a key role as cholesterol-lowering milk additive, which likely increases the immune responses, exert antimutagenic and anticarcinogenic activities and protect against gastrointestinal diseases, as summarized in numerous scientific review articles, e.g., (Nagpal et al., 2012; Kolaček et al., 2017). Several tools were introduced to monitor physiologic key parameters in *L. plantarum* cultivations like multi-parameter flow cytometry (Arnold et al., 2002; Schenk et al., 2008; Bensch et al., 2014; Tropcheva et al., 2015), quantitative real-time PCR, e.g., (Clementschitsch et al., 2005; Davis, 2014; Sohier et al., 2014; Pega et al., 2016; Emerson et al., 2017), and viable cell counting for viability analysis (Savini et al., 2010; Perdana et al., 2012; Glušac et al., 2015). Although all these systems provide information on the metabolic state and physiology of the cell, they require manual *off-line* sample pre-treatment. The necessity of sample pre-treatment might be the reason for the lack of correlation between results of flow cytometry and colony forming units of *Lactobacillus* sp. (Léonard et al., 2016). The electrooptical analysis of the anisotropy of polarizability (AP), in contrast, represents a fully automated method that has been developed to monitor the viability of rod-shaped microorganisms, since they orientate under the effect of an electrical field. This orientation is measured by the change of extinction from two orthogonal light sources. The extinction decreases in the direction of orientation and increases in the orthogonal direction in comparison to a chaotic orientation. The time, which is needed for re-orientation depends directly on the cells' polarizability (Bunin, 2002). This principle is combined with a continuous and automated sampling and sample preparation (i.e., cell suspension filtration, adjustment of cell concentration and of conductivity) coupled to a flow cell (Angersbach et al., 2006a,b; Junne et al., 2008). The method provides a spectrum of the AP, since various frequencies (kHz to MHz) can be applied in one measurement.

The cell polarizability, if determined electrooptically, is mainly evoked by the Maxwell-Wagner polarization. It relies on the accumulation of electric charge at the interface between two media of different electrophysical properties (Zhivkov and Gyurova, 2008; Gyurova and Zhivkov, 2009). The interface of the cytoplasm and the cell wall is the main origin of this cell polarizability in case of bacteria. The intracellular ion balance is changing during the course of a cultivation due to substrate consumption and product formation, eventual inhibitor accumulation and an unsuitable pH-value, among others. This has an impact on the Maxwell-Wagner polarizability.

The authors have described the electrooptical monitoring of (i) the switch from the acidogenic to the solventogenic phase in *Clostridium acetobutylicum* cells (Junne et al., 2008), and (ii) the development of the polarizability in *Escherichia coli* batch cultivations (Junne et al., 2010). The slope of polarizability vs. the frequency made it possible to differentiate between phases of dominating acid or dominating solvent production in *C. acetobutylicum* cultures. Metabolite fluxes as determined from *off-line* concentration measurements correlated well with the course of the polarizability. A strong relation between the development of the polarizability and the specific acetate synthesis rate in *E. coli* experiments was observed.

The aim of the present study is the investigation of the time course of polarizability of *L. plantarum* ATCC 2014 in batch and fed-batch fermentations in complex, industrially relevant medium and at certain disturbances like a low pH-value, substrate pulse and temperature alterations. The ability to identify and predict certain cultivation stages based on the AP is investigated, with a special emphasis put on lactate synthesis and carbon source consumption. The main question is whether fermentation phases with active cells with high metabolic turnover rates can be distinguished from weak cells with low energy generation capabilities by means of the electrooptical analysis. Additionally, the development of the AP during the regeneration (cultivation) of freeze-dried cells as starter cultures is investigated. The AP showed distinct differences, samples taken from the retardation and late stationary phase had a higher AP soon after they were resuspended, and finally had a higher growth rate. The AP is thus a meaningful parameter to identify suitable harvesting stages prior to freeze-drying for the further use as a probiotic compound.

## Materials and Methods

### Bacterial Strain and Media

The strain *L. plantarum* ATCC 2014 was used throughout this study. In all batch and fed-batch cultivation experiments, a 50% standard MRS medium (Carl Roth, Karlsruhe, Germany) was used, containing (per liter): 10 g glucose, 5 g peptone, 4 g beef extract, 2 g yeast extract, 0.5 g Tween 80, 1 g K_2_HPO_4_, 2.5 g sodium acetate, 1 g ammonium citrate, 0.1 g MgSO_4_, 0.025 g MnSO_4_. Media in pre-cultures were twice as concentrated. Two milliliter of antifoam 204 (Sigma-Aldrich Chemie GmbH, Steinheim, Germany) were added to reduce foam formation in the stirred bioreactor experiments.

### Cultivation Conditions

Five hundered microliter of *L. plantarum* cell suspension from a cryostock were used to inoculate 50 mL of pre-culture and grown at 34°C overnight without agitation. Eight milliliter of this pre-culture were used to inoculate 200 mL cultivations in an EloFerm bioreactor (EloSystems, Berlin, Germany) when it reached a pH-value between 3.6 and 3.8 and an optical density (OD_600_) between 4.2 and 5.2. Fifty milliliter of pre-culture were used for inoculation in case of 2 L cultivations in a KLF 2000 bioreactor (Bioengineering, Wald, Switzerland).

The pH-value was controlled at 5.8 by the addition of 1 M and 7.5 M NaOH solution in the batch and fed-batch bioreactor cultivations, respectively. The temperature was maintained at 34°C. The culture was gently stirred at 150 rpm during the initial batch phase. The liquid was sparged with nitrogen at a rate of 0.03 vvm and stirred with 200 rpm in the subsequent fed-batch phase. Continuous feeding of a solution, which contained 50% (v/v) of MRS medium and 50% (v/v) of a 440 g L^−1^ dextrose solution was started after the glucose of the batch phase was consumed. The feed reservoir was weighted to ensure appropriate feeding. A feed rate of 0.24 L h^−1^ was applied during the first fed-batch phase, while it was doubled during the second fed-batch phase. The oxygen (if there was any) and carbon dioxide content were quantified with the exhaust gas analyzer All-in-One (BlueSens, Herten, Germany).

### Concentration Analysis

Optical density at 620 nm was monitored with a photometer EloCheck (EloSystems, Berlin, Germany) in a flow cell connected to a reactor bypass with an optical depth of 2.2 mm for every 15 s. The biomass concentration was additionally determined *off-line* with appropriately diluted samples at 600 nm in an Ultraspec 2100 *pro* UV/Visible spectrophotometer (Biochrom, Cambridge, UK).

The amount of cells per mL, c_n_, was calculated following eq. 1 under consideration of the cell length as determined with the electrooptical measurement and as calibrated with captures of *L. plantarum* cultures under the microscope:

$$\begin{array}{l} {\text{c}}_{\text{n}}=\left(3.1\cdot {\text{OD}}_{600}/{\text{l}}_{\text{c}}^{1.33}\right)\cdot 10^{9} \end{array}$$

l_c_ represents the mean cell length.

Metabolite analysis (i.e., glucose and lactate) and amino acid quantification was conducted with HPLC analysis as described previously (Lemoine et al., 2015).

The dried cell weight (DCW) was determined as follows: 1 mL of *L. plantarum* samples were centrifuged at 15,000 rpm and 4°C for 10 min, washed with 1 mL of 0.9% (w/w) NaCl and centrifuged again. The supernatant was discarded, whilst the pellet was dried in an oven at 70°C for 24 h. The amount of the residual biomass was determined gravimetrically.

In case of freeze-drying experiments, samples were taken after 4, 6, 8, and 21 h of a 200 mL bioreactor cultivation. Twenty milliliter of sample broth was centrifuged at 8,000 rpm and 4°C for 15 min. The supernatant was discarded and the wet sample was transferred to round bottom flasks. These were stored at −20°C for 1 day and then lyophilized with a LyoQuest lyophilizer (Telstar® Life Science solutions, Terrassa, Spain) until dryness at 0.9 mbar and −50°C. The different samples were suspended individually in 50% MRS and allowed to grow at 34°C in shake flasks with gentle mixing.

### Determination of Cell Polarizability and Cell Length

A fully automated, commercialized sampling and analysis unit EloTrace (EloSystems, Berlin, Germany) was used to monitor the AP and cell length in a sampling interval of 15 min. Cells were separated from the culture broth by filtration through a cellulose filter of a pore size of 0.45 μm (Sartorius, Göttingen, Germany). The cell concentrate was diluted with distilled water of a conductivity of 5 μS·cm^−1^ to a final optical density of OD_600_ = 0.1 ± 5% prior to the electrooptical measurement. The AP was acquired in a measurement chamber at four different frequencies: 210, 400, 900, and 2,100 kHz. Detailed principles of the method were described elsewhere (Bunin, 2002). All AP values contain a scaling factor of 5·10^−31^ F·m^2^ for easier readability and comparison with other literature sources. The system was calibrated by microscopic analysis in order to correlate orientation and relaxation characteristics to the cell length.

### Data Fitting and Visualization

Data of OD_600_ measurements, which were obtained every 15 min or of DCW, which was measured every hour in triplicates, was fitted with the smoothing spline function in the curve fitting toolbox of MATLAB R2013b (The MathWorks, Natick, MA). The growth rate was then calculated for each time interval with the slope of the curve with logarithmic (ln) linearization of the OD_600_ or biomass time course. All data plots were created with SigmaPlot version 11.0 (Systat Software, San José, CA).

### Statistical Analysis

Data were expressed as mean standard deviation (SD) between duplicates for the description of reproducibility. Biological replicates were performed as fermentations under identical conditions.

## Results and Discussion

### Statistical Analysis of the Electrooptical Measurement

**Figure 1:**
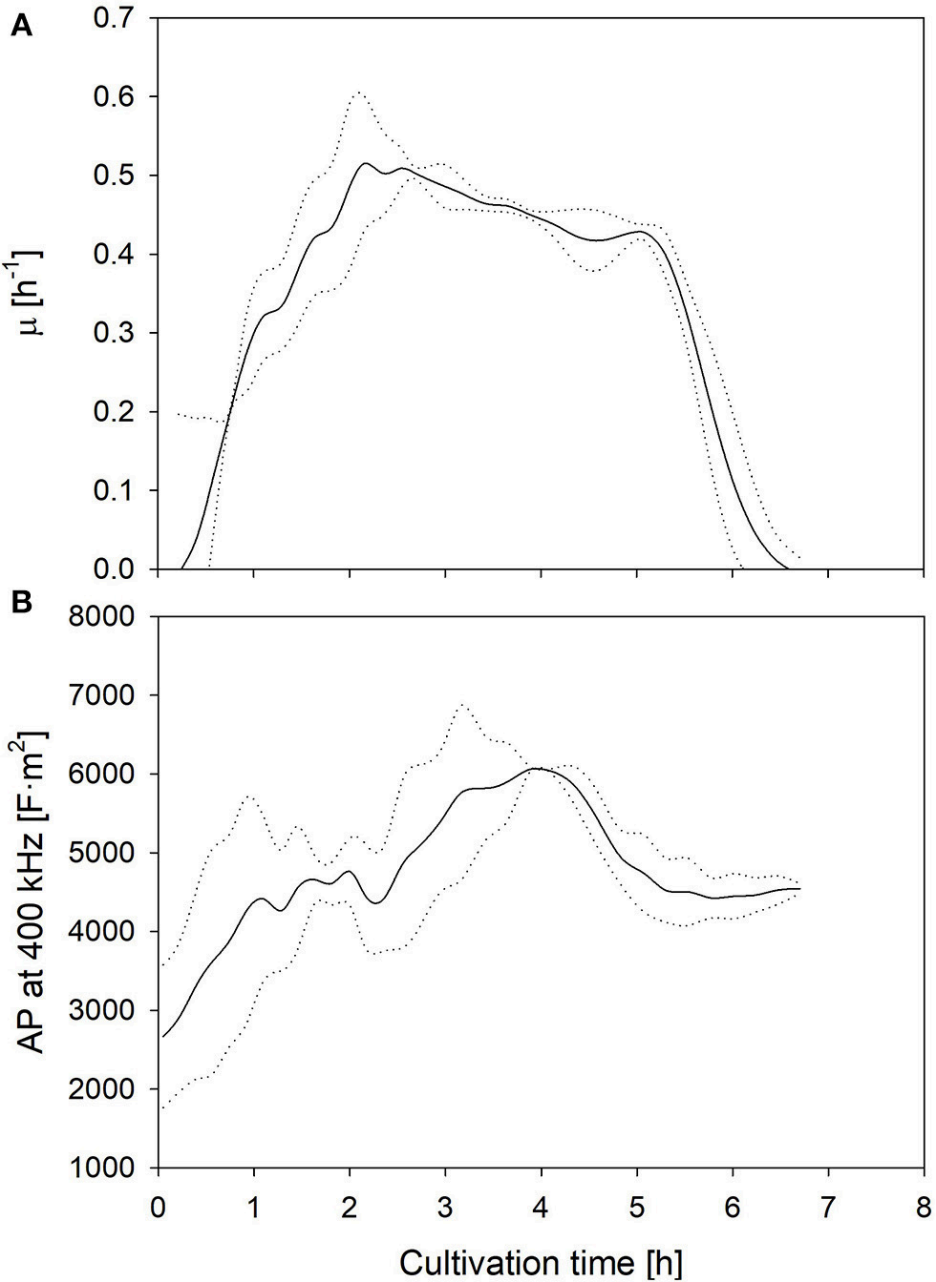
Mean growth rate **(A)** as well as AP level at 400 kHz **(B)** development during fermentations under optimum (standard) conditions. Dotted lines represent the limits of the standard deviations between two biological replicates.

The aim of this study is the investigation of the suitability of electrooptical polarizability measurements to determine uncomfortable growth conditions and potential losses of cell viability and metabolic activity. Therefore, the technical reproducibility of the measurement and the biological reproducibility of the physiology of the culture were determined. The biological reproducibility of the polarizability, measured in samples of two fermentations, which were performed under optimal growth conditions, is summarized in Figure 1. The mean specific growth rate and the AP at 400 kHz are shown together with the upper and lower limits of the standard deviation (SD). The values expressed a large deviance during the first hour due to a wider spread of the AP of pre-cultures at the harvest time, but the growth rate and AP profiles developed very similar afterwards. The duplicate measurements of the AP of the same samples yielded a deviance of 5% or lower at all samples. This encouraged further investigation whether different cultivation conditions will lead to a distinct change of the AP, and whether this change can be correlated with growth and lactic acid formation.

### Effects of Altered Cultivation Conditions on the AP and Cell Length

**Figure 2:**
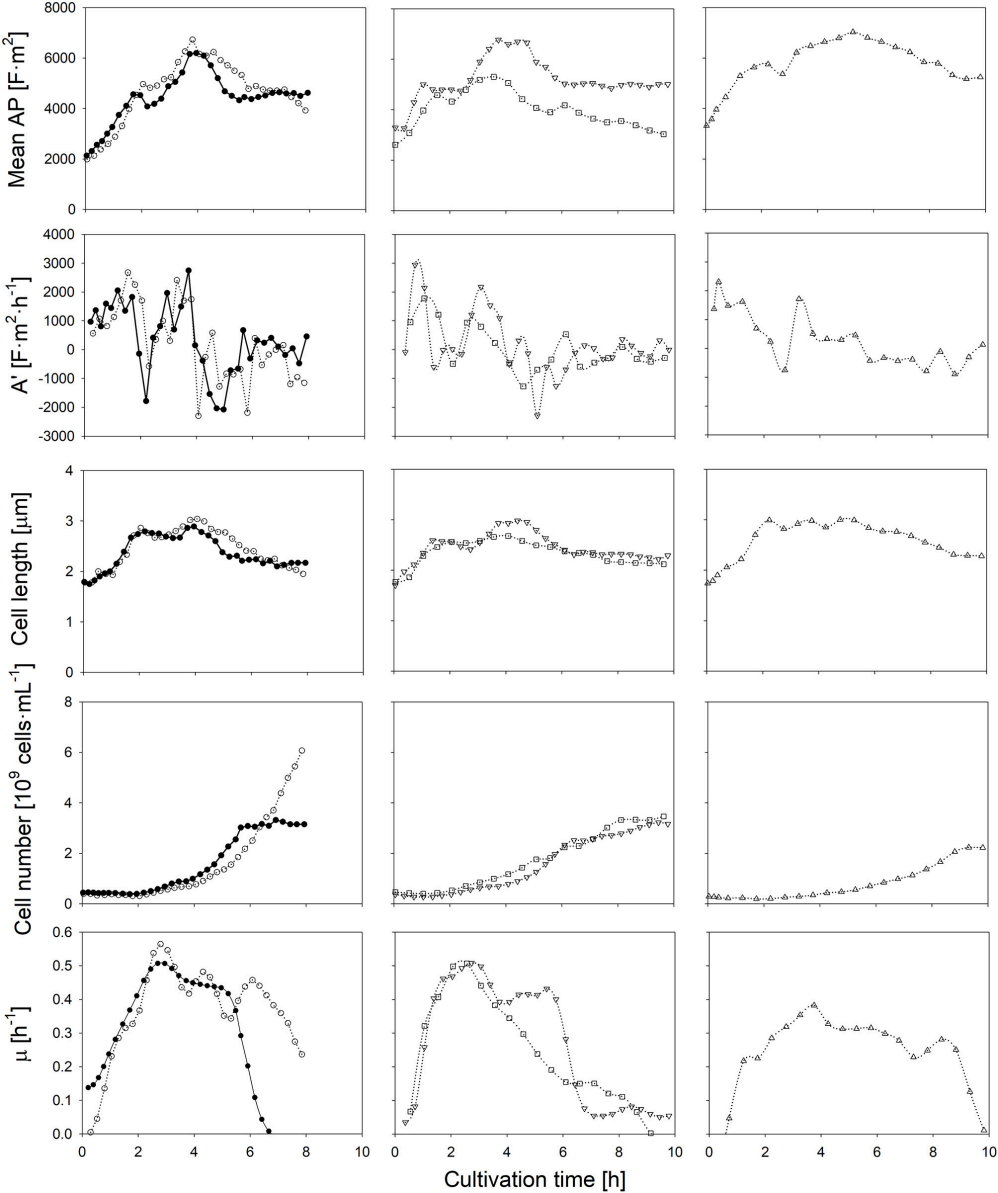
Time course of the mean anisotropy of polarizability (AP), its first derivate, cell length, the amount of cells and the specific growth rate throughout six *L. plantarum* batch cultivations: at standard conditions (•), with additionally 1% of glucose in the medium (◦), without pH control (□), with aeration (▿) and at a reduced temperature of 25°C (▵). Error bars depicting the technical error of the polarizability measurement have not been represented, thus enabling the comparison between cultivation conditions.

Firstly, in order to elucidate the impact of carbon availability, the initial amount of glucose was doubled in comparison to the control experiment, while it is assumed that the supply of ions and other essential nutrients remain unlimited (as observed from the growth rate). Secondly, fermentations w/o pH control were performed to investigate the influence of acidic conditions on the AP. Moreover, since the presence of oxygen may have an impact on the proton motive force in *Lactobacilli*, and thus the cells' polarizability, the nitrogen-sparged cultivation was compared to an aerated cultivation, in which a sparging rate of 1 vvm of air was applied. Finally, the impact of a lower cultivation temperature of 25°C, and thus a lower metabolic activity, on the AP was investigated (Figure 2).

If the initial glucose concentration was higher, the same AP profile was obtained at the beginning of the cultivation. The maximum growth rate was achieved when the AP reached about 5,000 F·m^2^. The AP decreased earlier if lower amounts of glucose were available. In both cases, the drop of the AP below the threshold value occurred within the same order and time as the growth rate declined during the course of the cultivation. As the specific lactate production was rather similar, the change of the AP cannot be attributed to lactate accumulation. Most likely, the earlier onset of substrate limitation at a lower initial glucose concentration was the reason for the changed AP profiles. The growth rate does not develop in parallel to the cell number in the beginning of the cultivations, as growth measured with optical density and gravimetrical biomass determination relies on the increase of the cell length.

If the pH-value was not controlled, a sudden drop of the AP was observed after 3 h. The external pH reached a value of 4.5 at that time (Figure S2). Such a low pH value is regarded as unfavorable, as growth reduction occurs in such an acidic environment (Giraud et al., 1991; van de Guchte et al., 2002). Indeed, a growth reduction was observed about 30 min later. While the pH-value in the medium decreased further to a value of 3.6, the AP declined continuously (Figure S2). A low internal pH value has an impact on the cell viability (Valli et al., 2006) as it reduces the internal proton motive force. It is known that such an effect changes the transmembrane potential, mainly due to a change of the internal Maxwell-Wagner polarizability (Gyurova and Zhivkov, 2009). Transmission electron microscopy analysis revealed evidence of structural distortions of the cell surface of *L. casei* at pH-values of 4.0 (Hossein Nezhad et al., 2010). Such changes would surely affect the AP as the bi-electric layer of cells is weakened by the structural changes of the cell wall. Additionally, the non-dissociated form of lactic acid (pK_a_ = 3.86) is present at higher concentrations under acidic conditions. A passive transport by diffusion into the cell increases the lactic acid stress in bacteria (Hansen et al., 2016). In this case, cells use their energy mainly to shield them against this stress in order to maintain homeostasis rather than growth.

In contrast to a low pH-value, air sparging retarded growth only slightly and had no negative impact on the cells' physiological state. The time course of the AP and mean cell length was rather the same as in the control cultivation. It was observed earlier that the presence of heme and NADH as electron donor supports a fully active respiratory chain and the evolvement of a sufficient transmembrane potential in *L. lactis* and in other lactic acid bacteria (Brooijmans et al., 2007; Lechardeur et al., 2011). In case cells grow on heme, oxygen consumption is conducted with membrane vesicles. In that case, proton release is conducted by the respiratory chain rather than by H^+^-ATPases (Blank et al., 2001; Pedersen et al., 2012). Due to the complex components in the industrial medium as it was used in this study, heme and NADH shall be present in excess at least at the onset of the batch cultivation, thus aerobic respiration was eventually activated. It was found that aerobic respiration at sufficient nutrient availability is even beneficial for the energetic household regeneration of *L. plantarum* (Guidone et al., 2013) despite to some common views. The AP, however, was obviously not altered although the proton motive force might have been affected.

Finally, at a lower fermentation temperature, the rate of chemical reactions is naturally lower, which yields a reduced specific growth rate and lower final cell number, as also observed for other lactic acid bacteria (Cheigh et al., 2002). The highest mean cell length among all experiments, however, is observed during a lower cultivation temperature. If the lower temperature reduces turnover rates in late reaction steps of the metabolism more profoundly than the substrate uptake, an intracellular accumulation of intermediates will occur. This leads usually to larger cell lengths due to a higher osmotic pressure inside the cell (Junne et al., 2010; Pilizota and Shaevitz, 2014). The temperature shift however did not alter the physiological conditions of cells notably. In this case, the consideration of both, the AP and cell length, can provide suitable information about conditions, in which a high growth rate can be achieved.

The *at-line* monitoring of the AP enabled the identification of suitable cultivation conditions for all cases: an optimal range of the AP and cell length can be assumed, in which the cell reaches an optimal physiologic and morphologic state, that is an AP of above 4,500 (that is 22.5·10^−28^ F·m^2^ if the scaling factor 5·10^−31^ is considered) at 400 kHz and a cell length of about 2.5 μm. Growth was always high during periods, in which the AP and cell length stayed above these thresholds. Both, the AP level at 400 kHz and the cell length showed a certain correlation with the specific growth rate (Figure S1). Since the AP changed earlier than growth rates, the correlation between it and growth values is not very strong. Nevertheless, the AP measurement is suitable to act as an early indicator for growth state changes. The AP and cell length allow the identification of different growth phases among all batch experiments: (i) an acceleration phase, during which the AP and the cell length increase rapidly as cells exhibit an increased metabolic activity; (ii) a log phase, during which the AP and cell length reach maximum values in parallel to the growth rate; (iii) a deceleration phase, in which the AP decreases as the cell lenght does while the growth rate steadily declines, and (iv) a stationary phase, during which bacterial growth is retarded and the AP and cell length remain almost constant.

**Figure 3:**
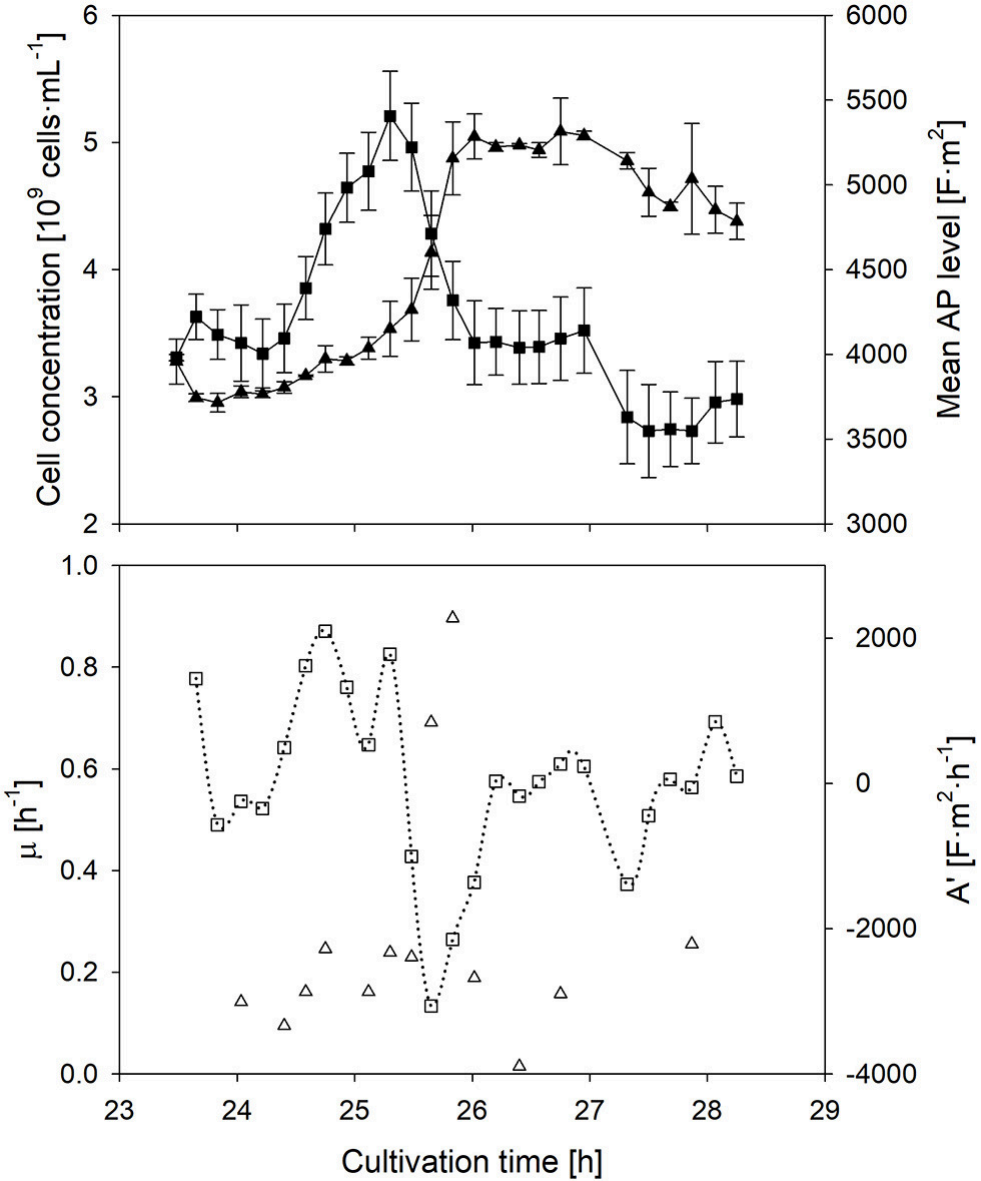
Time course of the mean anisotropy of polarizability (■), its first derivative (□), cell concentration (▴) and specific growth rate (▵) after a sudden substrate pulse after cells suffered 17 h of starvation. Error bars represent the technical reproducibility of EloTrace with two biological replicates.

The individual cells' AP depends on the transmembrane potential, and thus the ionic transport from the outside to the inside of the cell, which is related to the metabolic activity; this phenomenon has been described in literature (Geise et al., 2014). In order to prove the dependency between the metabolic activity and the AP, and to investigate the response time of the AP to a changed nutrient supply, a sudden glucose pulse addition in the stationary phase was performed (Figure 3). The glucose pulse led to an immediate response of growth. In this case, the AP is supposed to increase immediately as well. Indeed this is the case.

The response time of the AP to a situation, in which the activity of cells declines again while the added substrate is depleting, was investigated as well. In this case, the AP declined at the same time when nutrient limitation was reached after the pulse. The decline of the AP started ~30 min earlier than the decline of the growth rate. This observation after nutrient depletion seems to be conserved among all batch experiments of this study.

### Response of AP to Variable Feed Rates

In non-aerated cultivations, the decrease of byproduct formation, mainly carboxylic acids, can be a reason to conduct a nutrient-limited fed-batch cultivation. Since cells have a reduced substrate uptake due to the limited availability of a main nutrient component, mainly the carbohydrate source, a reduced accumulation of intermediates inside the cell occur, thus restricting byproduct formation to the necessity to regenerate the energy household.

**Figure 4:**
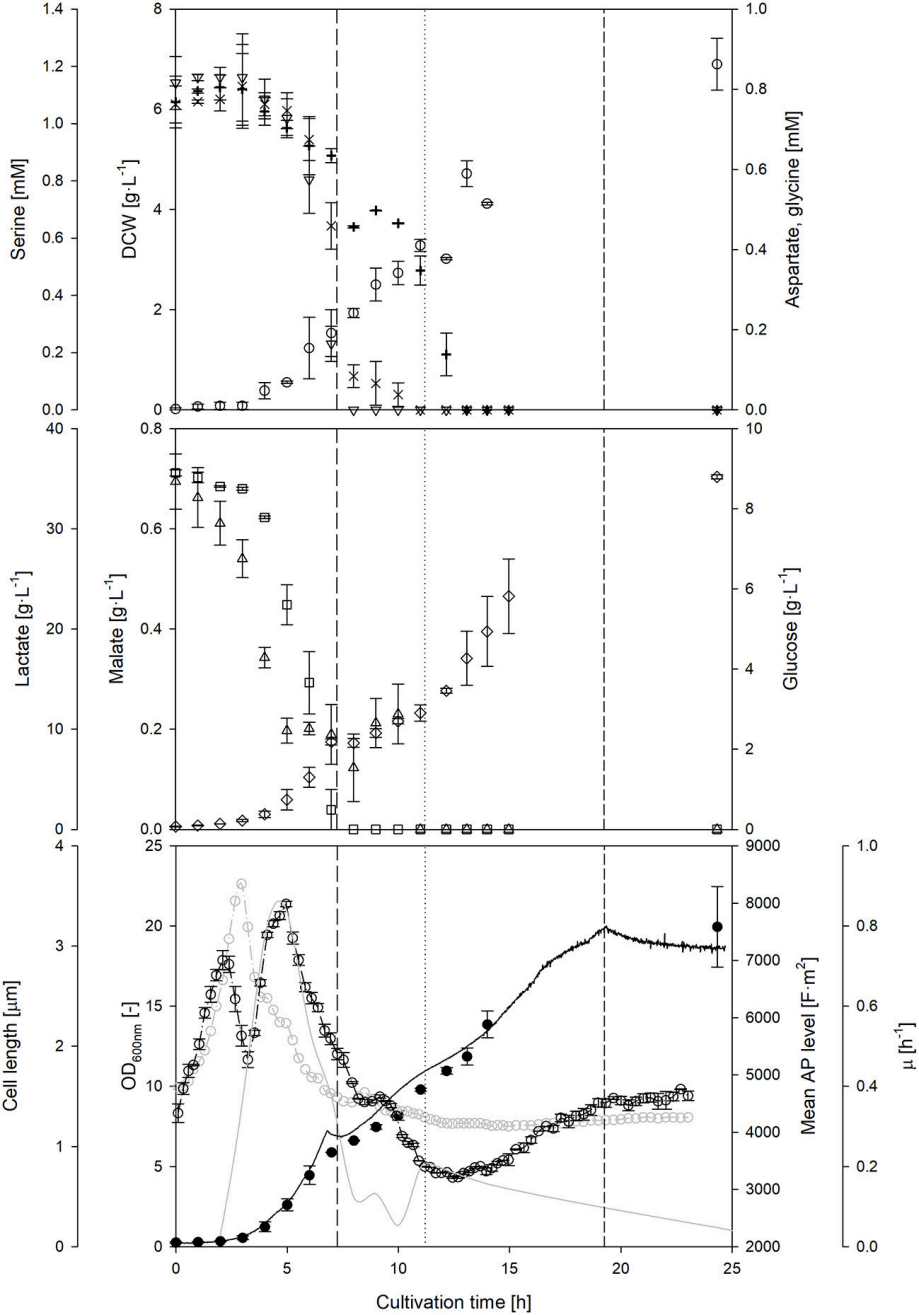
Course of *on-line* and *off-line* cultivation parameters, as well as main metabolites and amino acids throughout a *L. plantarum* fed-batch cultivation under standard conditions. *Off-line* monitoring of DCW (◦), aspartate (×), serine (▿), glycine (+), glucose (□), lactate (♢) and malate (▵) concentrations over time. *At-line* optical density (–•–), cell length (–◦–), mean polarizability (-−·◦−·-) and growth rate (–––––) measurements. During the fed-batch phase, the feed start (————), doubling of feed rate (…….) and feed stop (-------) are highlighted with vertical lines. Error bars denote standard deviation of two biological replicates.

The course of the AP was observed during a prolonged nutrient-limited growth phase (Figure 4). During a first fed-batch phase, the mean cell length remained almost constant, whereas the AP decreased. Due to the constant feed, substrate availability per cell is declining during this time, while the specific growth rate is reduced in parallel. Nevertheless, once the feed rate was doubled, a clear increase of the AP level was observed. These results confirm that the AP depends on the nutrient availability under nutrient-limited conditions, however, the dependencies were not as clear as in the batch phase. The second feed phase showed a rather decoupled development of the AP and the growth rate: in contrast to previous observations, the AP still increased when growth declined. Cells seemed to recover from the previous nutrient limitation through accumulation of intracellular components and restoration of cellular structures independently of growth. AP analyses under alternating nutrient-limited fed-batch conditions rather provide information whether feeding conditions are suitable to maintain or restore cellular structures rather than a direct correlation to metabolic activity. The AP remained rather stable after the feeding had stopped. It seems that the AP is hardly affected after nutrient depletion once cells adapted to nutrient limitation for a certain time before. This is an important observation if cells shall be kept for a longer time w/o nutrient supply: the time that is needed for adaptation might be observable with the AP measurement.

In order to explain some of the behavior of the AP during nutrient limiting growth conditions, the availability of other sources beside carbohydrates and carboxylic acids were investigated. A depletion of three amino acids was detected during the feed phases: firstly, serine and afterwards aspartate, and finally glycine. A sudden drop of the AP level seemed to occur at the same time when aspartate became strongly limited after 9–10 h of cultivation. Bacterial cells have a different preference, which amino acid they consume in dependence of the growth phase (Wolfe, 2005). It is assumed that the depletion of preferred amino acids in the medium will likely influence the AP, as cells either have to consume other amino acids or at least to synthesize the corresponding amino acids by themselves. This usually also changes intracellular fluxes and the energetic household. The AP might serve as an early indicator for amino acid depletion in the complex medium, however, this hypothesis requires further investigation.

**Figure 5:**
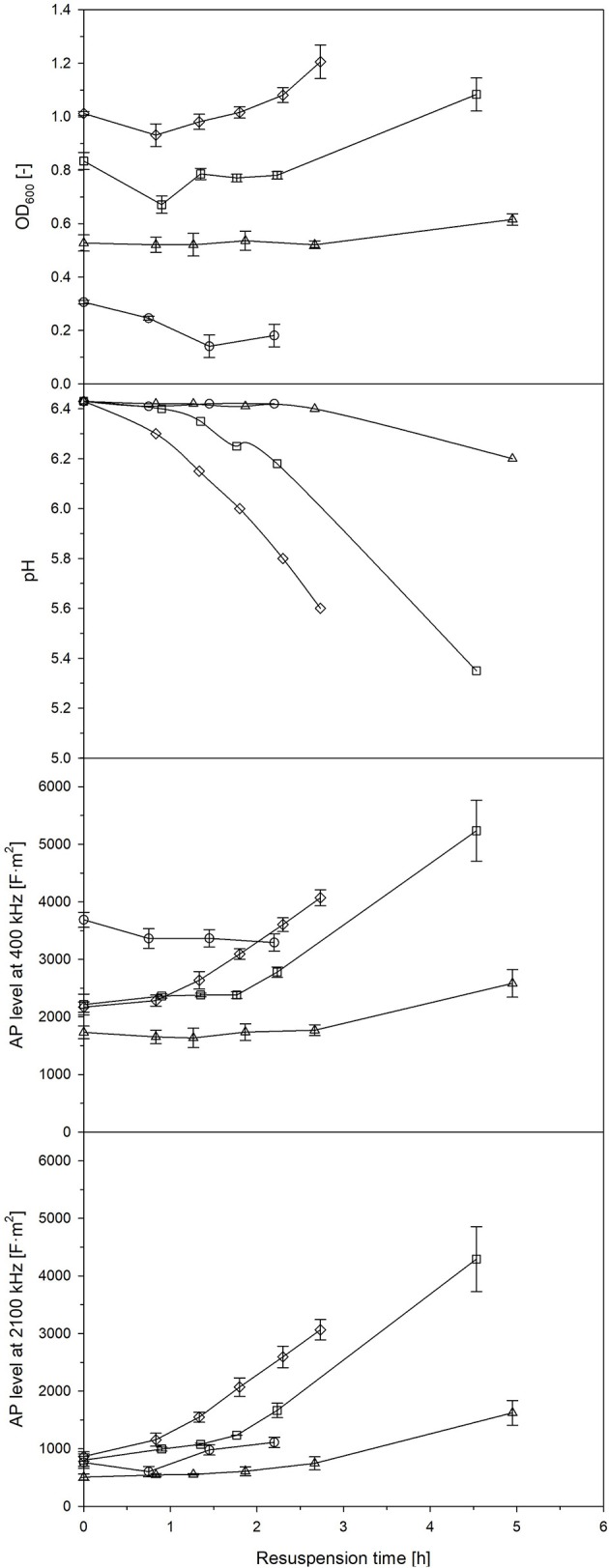
OD_600_, pH and AP level at a frequency of 400 kHz and 1,200 kHz of formerly freeze-dried and resuspended *L. plantarum* culture broth in 50% MRS media collected after 4 h (◦), 6 h (□), 8 h (▵) and 21 h of batch cultivation (♢). Error bars: Mean ± SD (*n* = 2).

*Streptococcus thermophilus* is used as proteolytic lactic acid bacteria in order to provide proteases to secondary microorganisms (Wu et al., 2015). For this purpose, lactic acid bacteria are usually dried, e.g., freeze-dried, and revitalized prior to use. The development of the AP after a longer phase of starvation was described in the previous section. Now, the behavior of the AP of *L. plantarum* after revitalization (resuspension) of a freeze-dried cell pellet was observed. Samples were taken at different phases of a batch cultivation (Figure S3): (i) during the growth phase, (ii) at strong retardation/growth cessation, (iii) in the early stationary phase 2 h after growth cessation, and (iv) in the late stationary phase 13 h later. Growth behavior varied, while the most profound growth was seen at samples taken at strong retardation/growth cessation and from the late stationary phase. These were also samples with the highest and fastest increase of AP after revitalization (Figure 5). If measurements at 400 kHz were compared with measurements at 2,100 kHz, a faster and more profound increase was seen at the latter frequency. It seems that a high frequency, in this case, is more suitable to distinguish between the different states of revitalization, e.g., the reconstitution of ion transport and functional cell structures. It was found that freeze-resistant cells had a high content of CH_3_ groups from lipid chains, cell proteins in an α-helix secondary structure and charged polymers, such as teichoic and lipoteichoic acids in *L. bulgaricus* (Passot et al., 2015). Certain options of drying cause membrane damages, which leads to high cell death (Bensch et al., 2014). Interestingly, the lactic acid production did not correlate well with the OD_600_, typical activity measurements based on lactic acid synthesis would have led to other results than growth and AP measurements, as the pH in samples taken at growth cessation decreased faster (that is a higher lactic acid synthesis) during revitalization than in samples taken from the late stationary phase.

## Conclusion

The *at-line* monitoring of the AP coupled to automated sample preparation enabled the identification of unfavorable cultivation conditions in *L. plantarum* batch and fed-batch fermentations. Different growth phases were identified throughout all experiments by means of electrooptical measurements. In contrast to many other studies with aerobic and anaerobic bacteria, *L. plantarum's* AP responded significantly to substrate pulses and insufficient substrate supply, and other unfavorable cultivation conditions. During fed-batch, the adaptation to nutrient-limitation is monitored with electrooptical measurements, thus providing a suitable parameter for optimization, as during revitalization after drying. The results support the hypothesis that an intact bielectric layer on the cells' surface yields a higher AP. Active cells with high metabolic turnover rates and energy generation were well distinguishable from weak cells with low regeneration capabilities, although the frequency matters for the distinguishability. In summary, the electrooptical monitoring represents a promising analytical tool, if conducted automatically as performed in this study, e.g., for the achievement of suitable production and conservation methods of *L. plantarum* and eventually other lactic acid bacteria.

## Author Contributions

KP-A conducted the experiments, collected data and wrote the manuscript. AA supported the electrooptical measurements and the preparation of the manuscript. PN supported supervision and the preparation of the manuscript. SJ supervised the work and the manuscript preparation.

### Conflict of Interest Statement

The authors declare that the research was conducted in the absence of any commercial or financial relationships that could be construed as a potential conflict of interest.
