# Supplementary material for: Electrooptical Determination of Polarizability for On-Line Viability and Vitality Quantification of Lactobacillus plantarum Cultures
Source: Front Bioeng Biotechnol. 2018 Dec 4;6:188. doi: 10.3389/fbioe.2018.00188 (PMC6289024; doi:10.3389/fbioe.2018.00188)

Supplementary material

Figure S1. Correlations between the specific growth rate (μ) and the AP level at 400 kHz (●) as well as the mean cell length (○) in the biological duplicates of *L. plantarum* batch cultivations under optimum conditions. The coefficients of determination are shown as R^2^ inside the plots.


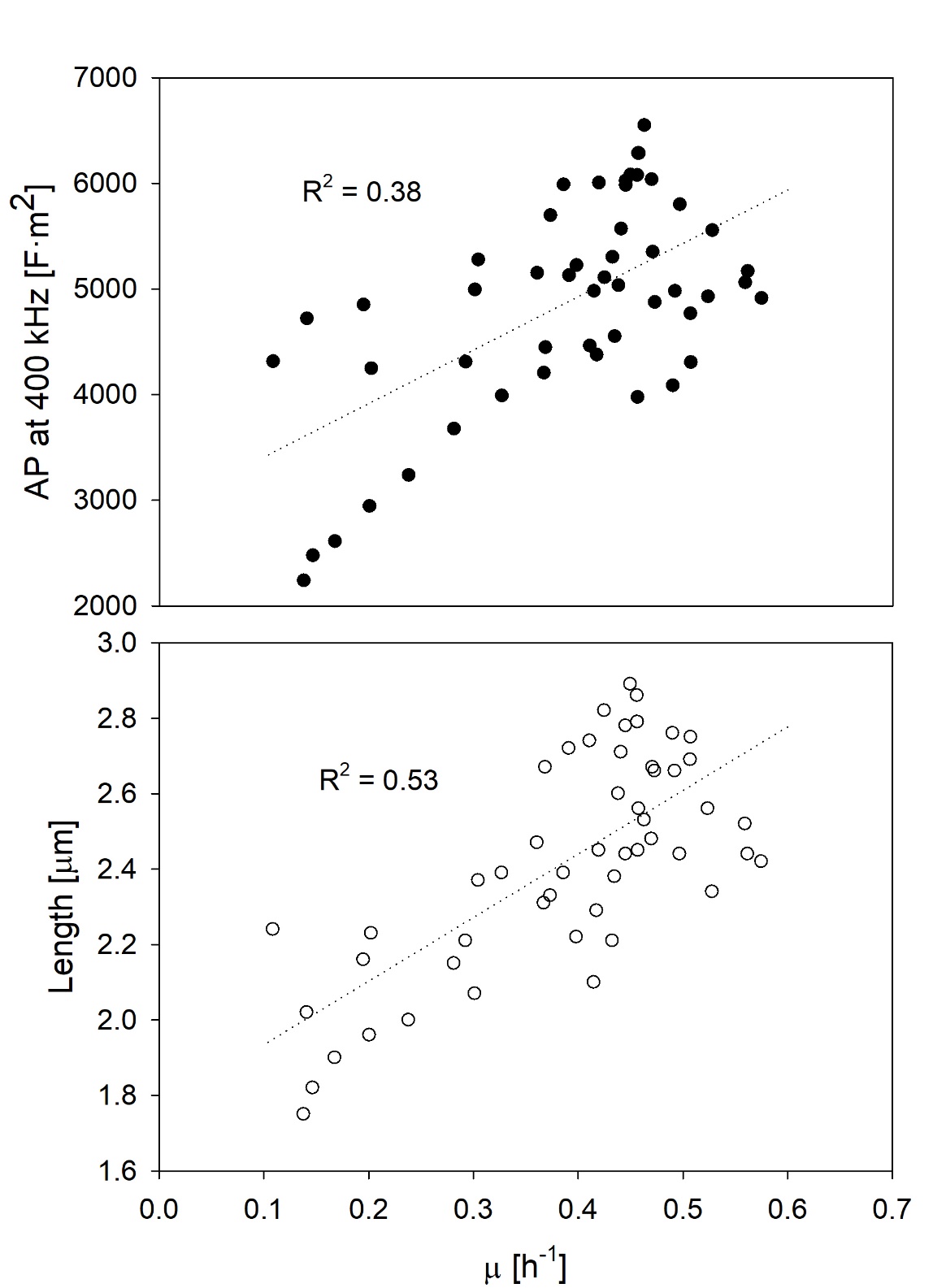


Figure S2. Development of the AP level at 400 kHz as a function of the pH of the culture broth.


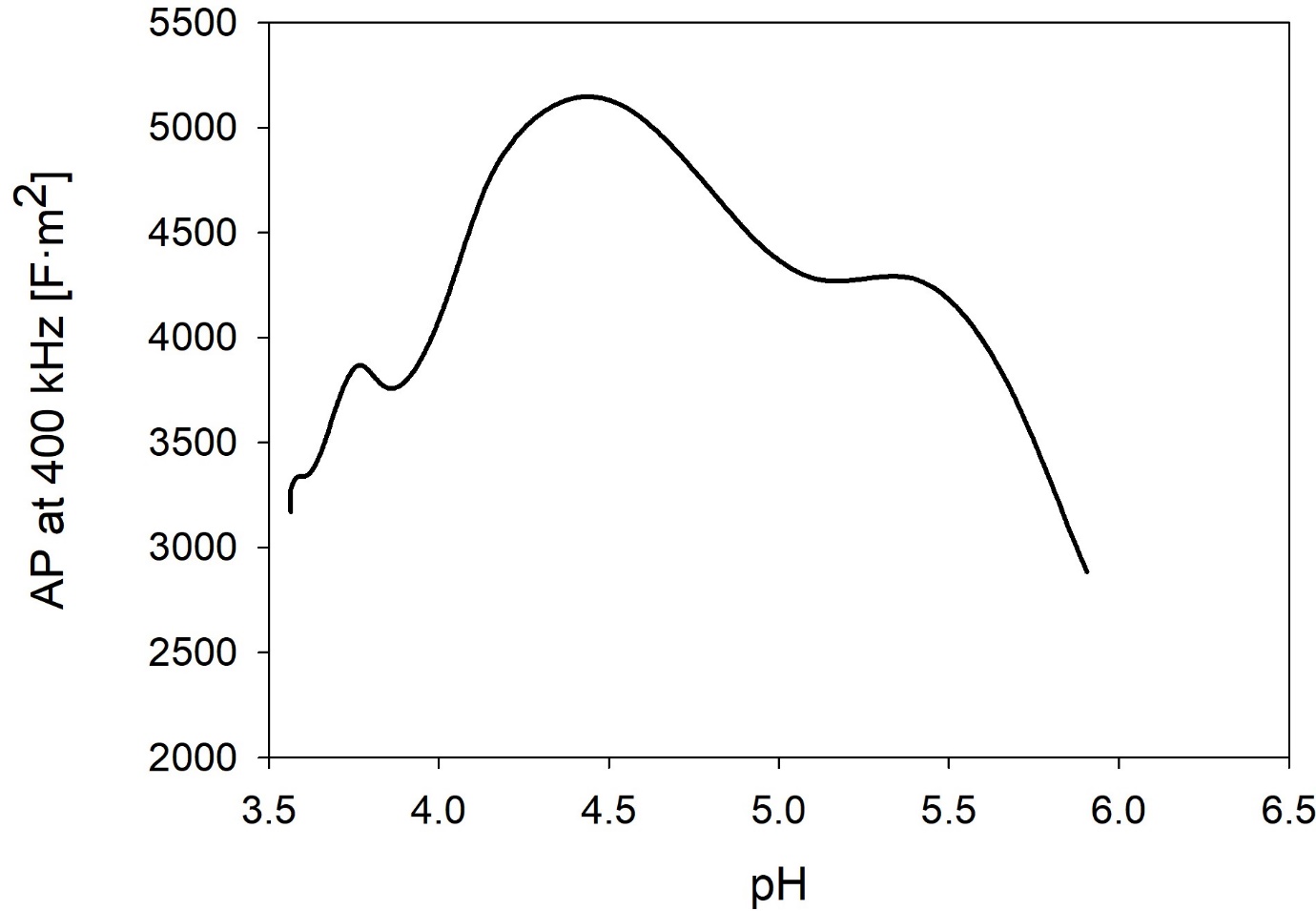


Figure S3. OD_600_ (black line), growth rate (grey line) and sampling points (short dashed lines) during a *L. plantarum* batch cultivation.


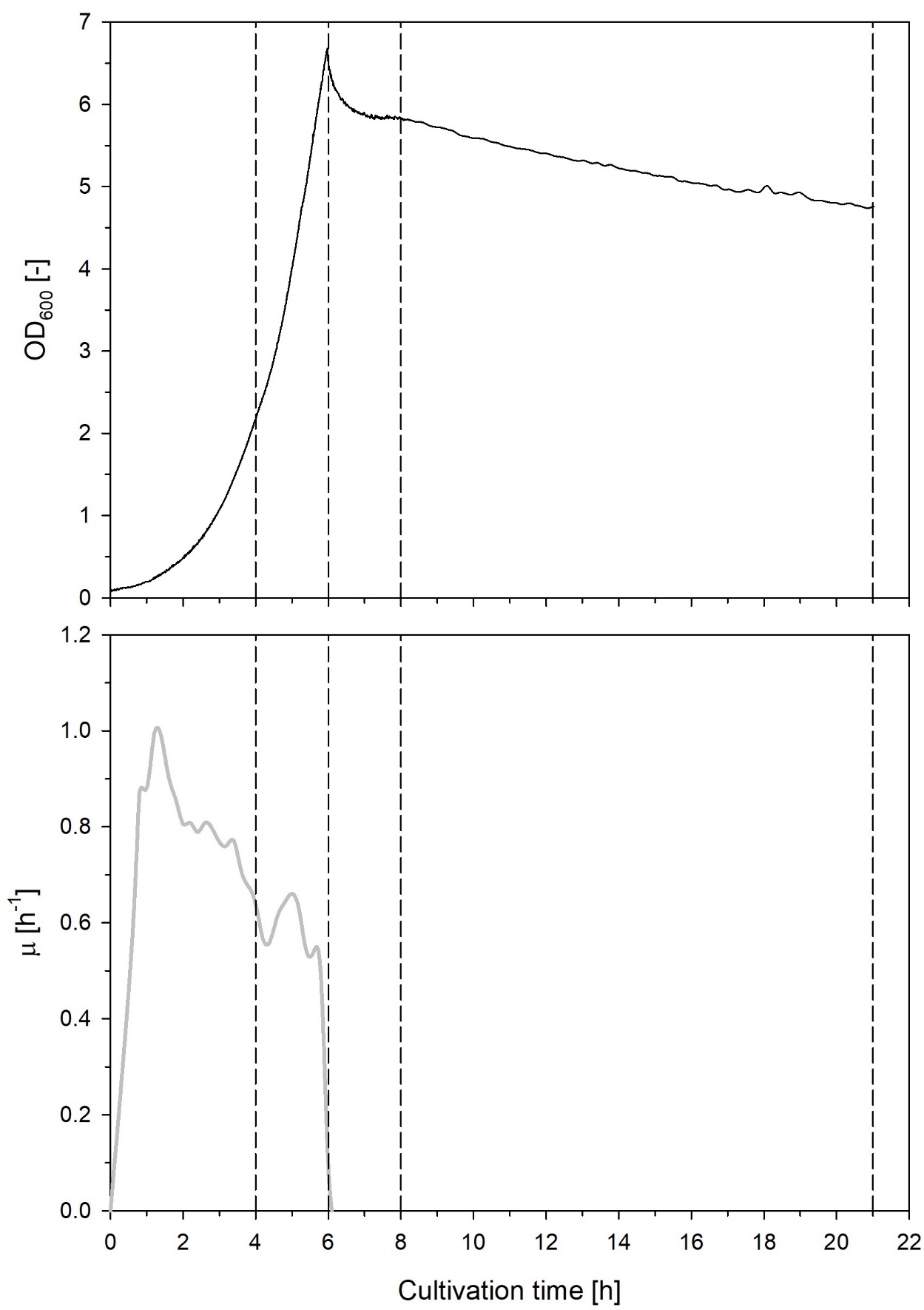

Supplement: Supplementary file 1 [file Data_Sheet_1.docx]
